# Recruitment and retention of farm owners and workers for a six-month prospective injury study in New Zealand: a feasibility study

**DOI:** 10.1186/1745-6673-6-16

**Published:** 2011-05-25

**Authors:** Simon Horsburgh, John D Langley

**Affiliations:** 1 Department of Preventive and Social Medicine, University of Otago, Dunedin, New Zealand; 2 Injury Prevention Research Unit, Department of Preventive and Social Medicine, University of Otago, Dunedin, New Zealand

## Abstract

**Background:**

Agricultural workers experience high rates of occupational injury. There is a lack of analytic studies which provide detailed occupational exposure information to inform intervention development.

**Methods:**

A feasibility study simulating a six month prospective cohort study was designed and undertaken. The levels of farm and worker participation and retention were analysed to determine the feasibility of the methods for wider deployment.

**Results:**

Recruitment levels were comparable with other studies, with 24% of farms and 36% of non-owner workers participating. Once recruited, retention was high at 85% and 86% respectively.

**Conclusions:**

The main challenges identified were in the recruitment process. Once recruited, farms and workers tended to complete the study, indicating that prospective studies in this the agricultural workforce may be feasible. Issues encountered and potential solutions for future studies are discussed.

## Background

Agriculture is widely recognised as one of the most hazardous industries in both industrialised and developing countries [1]. In New Zealand, agriculture is among the top three industries for fatal and non-fatal occupational injury [2,3].

Information available from descriptive epidemiological studies has highlighted potential avenues for reducing rates of injury in the agricultural sector [4,5]. However, in the early 1990s researchers noted a dearth of risk factor and detailed exposure information, and that this has hindered the development of properly informed injury control interventions [6-8].

One facet of this has been the collection of time-exposed information on occupational exposures. Much of the literature has used persons-exposed exposure estimates to calculate injury rates. While these can help with identifying exposures with high injury burden, they can be substantially incorrect when attempting to ascribe risk. This was demonstrated by Nordstrom et al. [9] when they compared the injury rate ratios for males versus females when calculated using persons-exposed and time-exposed denominators. Using persons-exposed denominators, they found a rate ratio of 2.4. This dropped to 0.9 when using time-exposed denominators. If the persons-exposed exposure estimate had been used, injury control resources may have incorrectly been targeted towards males on the basis that they were a higher-risk group.

There is an element of feasibility versus the ideal in the choice of collecting persons-exposed information. The agricultural workforce is difficult to access and measure, so there is a trade-off between what is feasible compared to what researchers would ideally like. That agricultural work is often long and demanding is well-documented [10,11]. This can make agricultural workers difficult to recruit and retain in analytic studies, particularly prospective studies which can have prolonged and/or demanding participation commitments.

The West Jutland Study (WJS) stands out as a potential model of a feasible prospective design for capturing detailed exposure information [12-14]. In that study, workers on pig farms were required to provide detailed time-exposed information on occupational activities and exposures every month for two years of the three year study period. The study was a trial of a safety intervention, and so required substantial commitment from participants. The researchers were able to initially recruit 59% of approached farms, with 51% of those completing the study [14]. These figures are comparable with other prospective studies in the agricultural workforce. Initial participation rates have typically ranged between 25% and 77%, with final participation rates ranging between 33% and 56% [15-19].

We conducted a feasibility study to determine whether a prospective cohort study modelled on the WJS with frequent, detailed exposure monitoring would be feasible in a different setting and encompassing a broader range of farm production activities. Our study also did not involve an intervention component and was substantially shorter (24 weeks). In this paper we focus specifically on the recruitment and retention rates achieved. We note that few studies have published their recruitment and participant retention methods in detail (see [18,20] for examples, however), and that none of these have been prospective studies requiring sustained active participation. We have therefore described the methods we used in detail. We do so here to help highlight possible barriers to the success of such studies, as well as the facets of the study methods which we think may improve the likelihood of success.

## Methods

### Design

The design of the feasibility study was modelled on the WJS, and simulated a prospective cohort study. A group of farm workers were monitored prospectively for a twenty-four week period. During this period they were required to provide information on the time engaged in specified work tasks, or working with specified animals and farm equipment. They were also required to report any occupational injury which affected their work pattern. Injury events were followed up with an in-depth telephone interview. Questionnaires were administered at the beginning and end of the study to obtain information about the workers and farms in the study, and to assess changes over the course of the study. Finally, a subset of farms were visited to compare worker descriptions of the farm environment with the observations of an independent assessor.

The recruitment phase followed a two-tier approach. Farm owners were approached first and asked if they would allow their farm to be involved in this study. If consent was forthcoming, any further workers on that farm were approached and invited to participate. This approach was adopted for pragmatic and ethical reasons. While the contact details for farm owners were easily obtained, as explained below, the contact details for farm workers were not. The farm owners themselves provided the most accessible source of information for farm worker contact details. Also, the feasibility study required information about the safety status of the farm environment as well as occupational injuries occurring on it. We considered it unethical to obtain this sensitive information about the farm workplace from workers without the owner's permission.

### Study Population

Farms engaged primarily (i.e. deriving 50% or more of revenue) in pastoral farming activities were the focus of this study. Previous New Zealand research has highlighted the large number of injuries associated with animals [21-24]. Targeting pastoral operations was considered to be an efficient means of maximising possible injury events occurring during the study, in turn allowing better assessment of the study methods. Any loss of generalisability from the study findings was considered to be negligible given that the majority (64%) of New Zealand farms were engaged primarily in pastoral farming (customised information request from Statistics New Zealand, 1999) and that, even though most farms are engaged primarily in pastoral farming, many also engage in other non-pastoral production activities such as cropping and forestry [25].

The study sample was drawn from the Waitaki Territorial Local Authority (TLA) in New Zealand. This area was chosen because it had a large number of farming units with most engaged primarily in pastoral farming, had a range of pastoral farming activities, included a range of terrain types and was geographically close to the research centre. Further inclusion criteria were applied on the farms and farm workers within the Waitaki TLA. Farms had to be at least 30 hectares in size and contactable by phone (either land-line or cellular), as much of the study contact was conducted by telephone. Thirty hectares was deemed to be the minimum viable size for economic self-sufficiency for a pastoral farm, and was also used to reduce the number of 'hobby farms' potentially included in the study.

Farm workers were defined as anyone contributing labour for an average of four or more hours per week directly to the economic output of the farm. This included working owners and unpaid family members who contributed labour to the economic output of the farm. Participants had to be aged sixteen or over.

### Identification of Farms

Contact and demographic information on farms in the Waitaki TLA was obtained from the AgriBase™ database, a national database of farm ownership, location and management in New Zealand owned and maintained by AgriQuality. AgriQuality is a private company providing quality-assurance services to the agricultural sector. The AgriBase™ stores details about each farm, including its location, the contact details of its owner, the farm's size and the number of stock units present. AgriQuality estimated 95% of farms in the Waitaki TLA were recorded in AgriBase™ prior to the study (Quenten Higgins, personal communication).

Farm owner contact details for farms fitting the study inclusion criteria were obtained from the AgriBase™ so that letters could be sent to the farm owners and recruitment calls made.

### Identification of Farm Workers

Unlike farms, there is no single source of information listing farm worker contact details. Details about workers on farms were obtained directly from the consenting farm owners.

### Recruitment

The recruitment phase consisted of four components, each of which will be described in turn.

#### 1. Generating Local Awareness of the Study

It has been suggested that providing advance warning of research activity in an area can improve study participation by generating interest among the local population [26]. During pre-testing with a small group of farmers, it was mentioned that farmers were often approached by telemarketers and businesses, and that these approaches were not welcome. Providing advance warning of the study was also intended to help prevent study recruiters being dismissed as one of these groups.

Letters explaining the study and that a person would be telephoning soon were sent to all owners of eligible farms in the Waitaki TLA. Advertisements briefly explaining that recruitment would be occurring were placed in local newspapers. One of the authors (SH) also attended local farming group meetings to promote the study and solicit feedback.

Feedback obtained from pre-testing on participation incentives indicated that farmers were not keen to receive 'trinkets' or 'cute' gifts, which they associated with commercial organisations trying to gain favour. They expressed preference for monetary incentives in the form of a prize draw or similar. A cash prize draw was therefore offered to participants who completed the study. First prize was NZ$500 cash, with two runner-up prizes of NZ$250 each. The prizes were mentioned in all of the above correspondences.

#### 2. Hiring and Training of Recruitment Staff

Rural residents from the Waitaki TLA were approached through informal community contact for the position of recruiter. We anticipated that using people from Waitaki with local knowledge and involvement would enhance recruitment. Three recruiters having considerable involvement in the Waitaki rural community were employed. One of the authors (SH) also participated as a recruiter.

All recruiters received training to familiarise them with the study objectives, protocols and record-keeping processes, and to develop consistent methods for dealing with potential issues using their knowledge of the local context. Meetings were held weekly for the first month and then fortnightly to discuss any issues which arose and to maintain consistency.

#### 3. Initial Telephone Recruitment of Farm Owners and Workers

Farm owners and workers were contacted by telephone. As suggested during pre-testing, telephone calls were made between 12:00 pm - 1:00 pm and 6:30 pm - 8:30 pm to coincide with when farmers would be at home for meals. Contacting farmers on Friday or Saturday evenings, or outside of these hours, was avoided unless invited. Messages were not left on answering machines during recruitment unless invited (such as when a family member initially answered the telephone and suggested we leave a message when we ring back).

### Recruitment of Farm Owners

We intended to contact all of the owners of eligible farms identified in AgriBase™. Each farm fitting the study inclusion criteria was given a unique randomly-assigned numeric identifier, and was contacted in that random sequence. Recruitment took place from mid-February through to the end of April 2002, a period of 2.5 months (the end of Summer and most of Autumn in New Zealand). A minimum of three attempts on a separate days were made to contact each farm owner. If a telephone number was invalid an attempt made to find the correct number through the telephone directory. Where the owner of a farm had changed, the current owner was asked for the listed owner's contact details and themselves invited to take part if their farm still fitted the study inclusion criteria. The originally listed owner was also contacted and invited to participate if their new farm fitted the study inclusion criteria.

Upon successful contact, the study was briefly described and verbal consent sought for the farm to be included in the study and workers on that farm contacted. Farm owners who did not allow their farms to participate were asked to complete a non-participating farm questionnaire over the telephone. This questionnaire was very short, and covered the following factors: production activity, dominant farm terrain, whether the farm had been profitable in the previous year, whether it had undergone a safety audit in the previous five years, the number of workers and residents on the farm, farm size and injury events in the previous year. The farm owner was also asked why they declined to participate. Farm owners who declined participation were asked if they could be re-contacted should the study not recruit a sufficient number of farms.

If the farm owner gave permission for their farm to be included in the study, the contact details for workers on that farm were obtained. The owner was also asked to participate if they worked on the farm.

### Recruitment of Farm Workers

Upon contact, the study was briefly described and verbal agreement to participate was sought. If the worker did not want to participate, they were asked to complete a very short non-participating worker questionnaire over the telephone. This questionnaire contained questions on the worker's age, gender, main farm job, years worked in farming, previous farm injuries, exposure to safety material and reasons for not participating in the study.

One person on a farm was designated the main contact for the farm. That person would be required to complete study items pertaining to farm-level information, as well as inform the research team if new workers started on the farm. Because this person would need to have a good knowledge of the entire farm and its running, the preference was to ask the farm owner or manager to take this role. If none of the workers from a farm agreed to participate or take on the main contact role, the farm was excluded from the study.

#### 4. Obtaining Written Consent from Farm Owners and Workers

All farm owners and workers who verbally agreed to participate were sent information and consent forms. The information forms provided detailed information about the study including an estimate of the time commitments for participation. The information and consent forms were designed according to local Ethics Committee guidelines, and differed slightly in content depending on whether they were for an owner or non-owner worker. The information sheets were four pages in length each. A covering letter explaining that the information forms should be read carefully and emphasising that a farm or worker could not start the study until their consent form had been signed and returned was included. Farm owners who were participating as workers were sent both versions of the forms.

A month was allowed for the return of consent forms. Telephone reminder calls were then made fortnightly until at least three reminder contacts had been made. If a consent form was not received from a worker after three reminder contacts, the worker was removed from the study. If a farm owner did not return the consent form for their farm, the farm and all of its workers were removed from the study. Likewise, if none of the workers from a farm returned a consent form the farm was removed from the study.

One of the farm contact's tasks was to notify the researchers if someone started or left working on the farm. This was intended to identify new workers on the farm so they could be recruited. However, no notifications of new workers were received and participants themselves notified the researchers if they were leaving the farm.

### Collection of Occupational Exposure and Injury Information

Participants were prospectively monitored for twenty-four weeks to collect information on occupational exposures and injury events. Each participant was initially required to complete a questionnaire to obtain baseline information about participant characteristics, occupational exposures and previous injury experiences. This questionnaire was eleven pages long, and took around twenty minutes to complete. The farm contact also completed a questionnaire containing questions about the farm's environment, previous injury history and work practices. This questionnaire was six pages long, and took around ten minutes to complete.

These questionnaires were followed by a twenty-four week monitoring period. At the end of every fourth week each participant was required to complete a written log of their work activities for the previous seven days and then post this log back to us. The log contained a list of common farm work activities. The participant ticked those they had performed and wrote down approximately how long they had spent on the task. A list of common farm items, including animals, was also included. The participant indicated whether and how long they had worked with these items. The log was six pages long and took five to ten minutes to complete.

During the monitoring period the farm contact was required to record any potentially work-related injuries occurring at the farm on a calendar. A broad definition of work-related injury was used, with a focus on capturing acute injuries which disrupted a worker's ability to perform their occupational duties. A work-related injury event was defined as 'any injury sustained through work activities related to the farm taking part in the study, even if off-site, and which resulted in: treatment from a health professional within a week of the incident, and/or restricted or impaired ability to perform work activities for four hours or more, and/or loss of consciousness'. The calendars were required to be completed weekly and then posted back to us every four weeks. Any participant reported as suffering a potentially work-related injury was contacted and interviewed about the injury event by telephone.

At the end of the monitoring period every participant was required to complete a final questionnaire. This questionnaire contained a subset of questions from the first questionnaire, and was used to assess changes in the participants and farm environment as well as solicit feedback on the study. The final questionnaire was ten pages long. The main contact was required to complete an additional questionnaire focusing specifically on the farm as a whole. That questionnaire was six pages long.

Farm site visits were performed on a random third of participating farms to assess the validity of reports of the farm environment. They involved a study team member visiting the farms, quantifying the items present of the farms and comparing their observations with what was recorded in the final farm questionnaires.

The farm owner's on-going consent was considered necessary for a farm and its workers to be included in the study. If the farm owner withdrew consent, then all workers on that farm were withdrawn from the study.

### Data Analyses

The recruitment and retention phases of the study were analysed separately. The recruitment phase was broken down into distinct stages so that points in the procedure where farms and workers were lost to the study could be identified. These stages were: initial telephone contact, verbal consent, and written consent. The retention phase was also broken down into stages for the same reason. These stages were: return of initial questionnaires, completion of the monitoring period and return of final questionnaires. Each of the stages in the recruitment and retention phases were analysed separately at the farm and worker levels.

## Results

### Recruitment into the Study

#### Farm Owners

A total of 611 farms were identified in AgriBase™ as potentially meeting the study inclusion criteria. Table 1 shows the number and percentage of farms at each point in the recruitment phase. Recruitment was slower than expected, resulting in only 307 (50%) of potentially participating farms being telephoned. Due to factors such as incorrect numbers and out-of-date information in AgriBase™, not all of these calls translated into successful contacts with the farm owners.

**Table 1:** Summary of the farm owner recruitment phase.

	*n*	%
Farms from AgriBase™	611	
*Initial Telephone Contact (% of Farms from AgriBase™)*		
Farms called	307	50%
Farms owners contacted	290	47%
*Verbal Consent (% of Farm owners contacted)*		
Farm owners consenting	144	50%
Farm owners declining	117	40%
Farms not fitting study criteria	29	10%
*Written Consent (% of Farm owners verbally consenting)*		
Farm owners consenting	70	49%
Farm owners not returning consent	8	6%
Farm owners changing mind	57	40%
Other reasons for not giving consent	9	6%

Approximately 50% of farms were lost to the study at each point of the recruitment phase. Of the 290 farm owners initially contacted, 70 (24%) actually consented in writing to their farms participating. Sixty-six (94%) of these owners also worked on the farm and agreed to participate themselves. The remaining four owners did not work on the farm.

Table 2 lists the main reasons given by farm owners for changing their decision. The most common reason by far was 'too busy.'

**Table 2:** Reasons given by farm owners for withdrawing consent to participant after verbal consent.

Reason	*n*	%
Too busy	27	47%
No longer interested	10	18%
Too onerous	4	7%
Ill health	3	5%
No reason given	9	16%
Other	4	7%
*Total*	*57*	*100%*

When the farm owners who verbally declined participation were asked if they could be re-contacted should more farms be needed for the study, 36 (31%) indicated that this would be acceptable and they would reconsider their initial decision at that point.

#### Non-Owner Workers

Table 3 summarises the number and percentage of workers available at each point of the recruitment phase. The main loss of workers occurred when written consent was requested, with 59% of the workers who had verbally agreed to participate not giving written consent. Ultimately, 28 (41%) of non-owner workers contacted provided written agreement to participate. The 'other reasons for not giving consent' category includes twenty-one (30% of those verbally consenting) workers who were lost to the study because the owner withdrew consent for the farm. This was higher than the number of workers who actively withdrew.

**Table 3:** Summary of the non-owner worker recruitment phase.

	*n*	%
Workers identified	80	
*Initial Telephone Contact (% of Workers identified)*		
Workers contacted	78	98%
*Verbal Consent (% of Workers contacted)*		
Workers consenting	69	88%
Workers declining	9	12%
*Written Consent (% of Workers verbally consenting)*		
Workers consenting	28	41%
Workers not returning consent	4	6%
Workers changing mind	11	16%
Other reasons for not giving consent	26	38%

### Selection Bias in Recruitment

Selection bias in farm recruitment was assessed by comparing responses from the initial questionnaires about farms with a subset of questions asked to farm owners who declined permission for their farms to participate. Sixty-nine (60%) of the farm owners who declined to give permission answered the set of questions. A similar process was used to assess selection bias in non-owner farm worker recruitment but only four workers who declined to participate agreed to answer further questions.

Table 4 shows the comparison between participating and non-participating farms. The main production activity was the production activity from which at least 50% of the farm's income was made. Mixed production farms had no activity which met this criterion. Differences were found between participant and non-participant farms, with non-participant farms tending to occupy high/mountainous (13% versus 6%) or flat terrain (15% versus 5%) compared to the rolling country of participating farms (39% versus 60%). They were also less likely to have recorded a profit in the previous year (72% versus 84%), undergone a safety audit in the previous five years (20% versus 89%) or had an occupational injury event on the farm in the preceding year (6% versus 31%).

**Table 4:** Comparison of participant and non-participant farms. Numbers in parentheses indicate the number of farms in each category after missing or erroneous responses were removed.

	Participant Farms		Non-Participant Farms	
	
	*n*	*%*	*n*	*%*
**Main Production Activity ***(n = 54, 66)*				
Dairy	3	6%	8	12%
Sheep	40	74%	46	70%
Beef	4	7%	6	9%
Deer	4	7%	0	0%
Mixed	3	6%	6	9%
**Terrain ***(n = 56, 61)*				
High/Mountainous	4	6%	8	13%
Hilly	11	18%	7	11%
Gentle/Rolling	37	60%	24	39%
Plains	3	5%	9	15%
Other	1	2%	13	21%
**Profit in Previous Year ***(n = 62, 69)*				
Yes	52	84%	50	72%
No	6	10%	14	20%
Did not wish to disclose	2	3%	5	7%
Did not know	2	3%	N/A	
**Safety Audit ***(n = 62, 69)*				
Yes	55	89%	14	20%
No	3	5%	53	77%
Did not know	4	6%	2	3%

	***n***	***per farm***	***n***	***per farm***
	
**Workers ***(n = 57, 69)*				
Total	130	2.3	121	1.8
**Residents ***(n = 62, 69)*				
Total	228	3.7	210	3.0

	***median***	***quartiles***	***median***	***quartiles***
	
**Size ***(n = 58, 69)*				
Hectares	282	214 - 695	231	98 - 950

	***n***	***%***	***n***	***%***
	
**Injury in Previous Year ***(n = 62, 69)*				
Farms reporting injury	19	31%	4	6%
	***n***	***per person/event***	***n***	***per person/event***
Injury events	23	1.4	5	1.2
People injured	21	0.9	4	0.8
	***rate per 100 farm-years***	***95% CI***	***rate per 100 farm-years***	***95% CI***
Injury events	38.3	27.1 - 51.0	7.2	3.1 - 15.9

### Retention Through the Study

#### Farms

Table 5 summarises the retention of farms throughout the remainder of the study. Thirteen farms (19% of those with written consent) in total dropped-out of the study before its end. The point of highest attrition occurred when sending out the initial questionnaires. Of the eight farms which were lost to the study at this point, two had been withdrawn by their owners, one was withdrawn because a farm-level initial questionnaire was not received back and another because the owner could no longer be contacted. The remaining four were removed because their farming activities were found to no longer meet the study inclusion criteria. All of the farms from this point on had owners who also participated in the study.

**Table 5:** Summary of the farm owner retention through the study (% loss from previous point).

	*n*	%
Written consent obtained	70	
Completed initial questionnaire	62	11%
Completed exposure/injury monitoring	60	3%
Completed final questionnaire	57	5%
*Total loss*	13	19%

Three of the five farms lost after an initial questionnaire was completed were withdrawn because the owner-worker on that farm simply stopped returning study materials.

#### Workers

Table 6 summarises the retention of workers throughout the study. Note that the initial figure of 97 workers is not the sum of the number of workers consenting and the number of farm owners consenting, as not all farm owners worked on the study farms. Just over a quarter of the workers who provided written agreement to participate had dropped-out of the study before it ended. As with farms, the point of highest attrition was with receiving the initial questionnaires back. All except two of the fifteen workers (87%) lost to the study at this point were lost because of the corresponding farm dropping-out of the study.

**Table 6:** Summary of farm owner and worker retention through the study (% loss from previous point)

	*n*	%
Written consent obtained	97	
Completed initial questionnaire	82	15%
Completed exposure/injury monitoring	78	5%
Completed final questionnaire	72	9%
*Total loss*	25	26%

Overall, most (75%) of the workers who dropped-out of the study were the owner of farms lost to the study or worked on such a farm.

### Participant Feedback on the Study

Participant feedback on the study was solicited through two items in the Final Participant Questionnaire, where they were asked to report any issues they had with completing the study and to suggest any improvements. Only fourteen participants recorded issues with the study. The primary issues identified were with completing study items in a timely manner (six participants) and some items being irrelevant to the participant's farming activities (five participants). None of the comments suggested that the study was onerous or cumbersome.

Participants' thoughts on the study were discussed informally as part of the farm site visit. No participants had negative feedback on the study requirements, and some expressed surprise at how little work it was.

## Discussion

### Recruitment

#### Farm Recruitment

Other research in New Zealand and overseas, including surveys or telephone interviews, suggest that a participation percentage of between 25 - 77% of farm owners contacted could be expected, with most falling within the 30-50% range [14-19,21,25,27-30]. Only 24% of contacted farm owners in this study ultimately participated in the monitoring part of the study. Several possible reasons for the low participation rate were identified. The first was the timing of recruitment, with farm owners being contacted in the New Zealand Summer and Autumn. Many farm owners were busy with hay- and silage-making for the Winter or planning holidays. This is likely to have reduced their willingness to participate and highlights the importance of performing recruitment at 'quiet' parts of the farm year when farm owners are unlikely to be considering holidays. This was consistent with the experience of Tarone et al. [18], who found that responders in their study of farmers were more likely to have enrolled in Winter.

The most surprising point of attrition in the recruitment process was at the written consent stage, given that verbal agreement to participate had already been obtained. The written consent forms (including study information sheets) were designed to strictly adhere to the guidelines provided by the local ethics committee. They gave the worst-case scenario for the level of time and involvement required when participating in the study, and were long and exhaustive in detail. This pessimistic view of the level of commitment needed from participants was likely to have given a bad impression to a population with a self-professed dislike of paperwork. The requirement to complete and return consent forms by post independently of other study items may also have been a deterrent. It is interesting to note that informal feedback from participants at the end of the study indicated that they did not think the study was actually that onerous. This suggests some advocacy from those who have participated may assist participation.

A further impediment to participation may have been that the study materials were predominantly paper-based. Techniques involving personal visits to farms or direct measurement were considered too resource-intensive and infeasible for a large-scale prospective study or on-going surveillance. This left methods based primarily on telephone contact or the Internet. Both of these methods were considered to be less suitable than postal methods for the present study. For example, internet penetration and usage within the rural sector of New Zealand at the time of this study was not high, with one study finding less than 40% of farmers used the internet for more interactive activities than basic web surfing and email (Unpublished data from a Ministry of Agriculture and Fisheries survey in 2001). The use of the internet as a research tool for the agricultural workforce should be evaluated for feasibility in the near future, however. In New Zealand, the government has signalled its intention to improve internet penetration and speed, particularly to rural areas. Similarly, progress in telephone-based techniques such as Interactive Voice Response (IVR) systems means that telephone administration of study materials may be more feasible than when this study occurred (see [31,32] for critical analyses of IVR systems).

#### Worker Recruitment

The final recruitment rate of non-owner workers was 36%, which was slightly better than that for farm owners but still low. If worker participation had not been dependent on the continued consent of the farm owner, the recruitment rate could have been as high as 50%. This highlights the weakness of the two-tiered approach to recruitment.

#### Bias in Recruitment

Differences were found between the farms where the owner gave verbal consent and those where the owner did not. These findings suggest that profitable farms with an interest in farm safety may be more likely to participate in studies of farm safety. The occurrence of an injury event on the participating farms may have increased safety awareness in these better resourced farms, leading to safety audits and interest in participating in studies such as this one. This was corroborated by the majority of participants reporting safety performance as their main reason for taking part in this study.

### Reasons for participation

As part of the initial participant questionnaire, participants were asked their reason for participating in this study. Fifty-six percent responded that they were participating to help increase safety on farms or simply to be helpful. A further ten percent reported participating because they thought the study might be interesting and might allow them to assess their own safety performance. Other responses (18%) consisted primarily of comments similar to 'you asked me' and 'wife told me'.

These comments show a high level of interest in farm safety among the study participants. This may indicate a bias for more safety-conscious farm owners and workers to participate, but may also suggest that pitching the potential safety benefits of research to the farming community, and potential participants in particular, may be a way to increase participation rates. Care would have to be taken with this approach, as it may exacerbate any bias due to more safety-conscious farm owners and workers participating.

### Retention of Farms and Workers

Retention of farms and workers throughout the study was good at 85% and 86% respectively between written consent and completion of the final questionnaires. This compares favourably to retention rates from other prospective studies of between 33% and 47% [14-19]. The main threat to retention was not the withdrawal of owners or workers from the study, but selling off part or all of the farm and therefore no longer meeting the study inclusion criteria. The low number of farms and workers withdrawing during the study precluded identifying common factors among them. This finding highlights the potential for the composition of the agriculture sector to change reasonably quickly in response to the economic environment, which can make lengthy research challenging.

Retention might have been worse if those farms and workers who dropped out at the written consent stage had not done so. The onerous consent process may have actually improved retention by filtering out less committed participators. However, our study does provide some evidence that if farm owners and workers are successfully recruited, they are likely to complete the study, and that detailed longitudinal data can be obtained from farms.

### Generalisability

Only primarily pastoral farming operations were included in this study. The study was also limited to a specific area. However, while Table 4 implies a certain level of homogeneity in the farm production activities, in actuality most farms were engaged in several production activities (mean = 2.1, *SD *= 0.7), including crop farming. There was also a reasonable mix of farming environments and sizes.

## Conclusions

The low number of participants recruited imposed some limitations on the conclusions which could be reached. Nevertheless, we feel that there are some useful conclusions and insights which may be of benefit to other researchers in this area.

This study highlighted the importance of making the consent process as streamlined and easy for participants as possible. This might seem obvious, but our observation was that the agricultural community is very averse to even moderate amounts of reading and form filling. The information and consent forms should be kept as short as possible, and convey information about what the study will realistically require of the participant rather than just the maximum commitment. Giving the participants the option of providing verbal final consent (through voice recording, for instance) or including written consent in the initial study items could also improve recruitment rates.

The process used by the recruiters did not lead to as many recruitments as expected. Part of this was due to the time required to make contact with farm owners and workers. While participant identification and recruitment is often a protracted and intensive process even in non-prospective analytic studies [20], it was surprising how difficult it often was to contact farm owners and workers. Despite calling at times suggested to us during pre-testing and by contacted owners themselves, many calls were often required to contact an individual when they could discuss the study. Recruitment calls also often took longer than anticipated due to the people contacted wanting to discuss farming-related matters with the recruiters. For studies requiring a large number of participants, we would recommend using a large number of recruiters during the less busy period of the agricultural calendar, such as Winter. We would also recommend against attempting to shorten recruitment calls. The conversations occurring during these calls can be seen as an important part of building a relationship with the participant community. Having recruiters who are able to discuss farming matters, as was the case in this study, would also be beneficial.

Once participating in the study, participants were unlikely to stop participating. This suggests that extended studies which obtain in-depth exposure and injury event information are feasible in the agricultural workforce. However, the high retention rate may be an artefact of less committed participants dropping out at the written consent stage.

It was apparent that attributes of the farms taking part could change significantly, even during the relatively short time period of this study. While these changes only led to minor losses of farms and participants from this study, they could be a serious issue in other studies. In particular, research aimed at more specific subgroups within the agricultural workforce may be particularly susceptible to this issue. A downturn in dairy prices might seriously compromise a study on dairy workers, for example, as farmers respond by changing the production mix of their properties away from dairy.

## Competing interests

The authors declare that they have no competing interests.

## Authors' contributions

SH conceived of the study, led its design, carried out the data collection and analyses and drafted the manuscript. JDL participated in the conception and design of the study, supervised the data collection and analyses and commented on manuscript drafts. All authors have read and approved the final manuscript.
